# Asymmetric Presentation of Corneal Ectasia With Acute Hydrops 34 Years Following Bilateral Radial Keratotomy

**DOI:** 10.7759/cureus.82884

**Published:** 2025-04-24

**Authors:** Aya Hiiragi, Hideki Fukuoka, Minori Minamide, Koji Kitazawa, Chie Sotozono

**Affiliations:** 1 Department of Ophthalmology, Kyoto Prefectural University of Medicine, Kyoto, JPN

**Keywords:** acute hydrops, corneal ectasia, keratoconus, radial keratotomy, refractive surgery complication

## Abstract

Despite the subsequent emergence of more advanced refractive surgical techniques, ophthalmologists continue to encounter patients who have experienced long-term complications from radial keratotomy (RK). A notable complication of RK is corneal ectasia, which, although rare, can lead to significant vision impairment years after the procedure. The present case report details a 54-year-old male patient who developed unilateral corneal ectasia with acute hydrops 34 years following bilateral RK. The patient exhibited symptoms including decreased vision, corneal opacity, thinning, and steepening in the right eye, while the left eye demonstrated only mild corneal changes. Notably, the development of acute hydrops with Descemet's membrane rupture was observed, mimicking the pathophysiology characteristic of keratoconus. A comprehensive discussion of the patient's clinical course, imaging findings, and management is presented, along with a review of the current treatment options for post-RK ectasia. This case underscores the necessity of prolonged follow-up for patients who have undergone RK, emphasizing the importance of vigilance for this uncommon complication, which can manifest decades after the initial procedure.

## Introduction

Radial keratotomy (RK) was first introduced in the 1970s by Fyodorov in the Soviet Union as a surgical procedure to correct myopia. The procedure entails the creation of radial incisions in the cornea, typically ranging from 4 to 24 in number, with a depth that reaches approximately 90% of the corneal thickness. The purpose of these incisions is to weaken the biomechanical stability of the cornea, causing the central cornea to flatten and thereby reducing its refractive power [1].

Although RK was widely performed through the 1980s and early 1990s, it has since been largely replaced by more predictable and safer procedures such as laser in situ keratomileusis (LASIK), photorefractive keratectomy (PRK), and small incision lenticule extraction (SMILE). Nevertheless, ophthalmologists continue to encounter patients who underwent RK some decades ago and now present with complications.

One of the rare but serious long-term complications of RK is corneal ectasia (progressive thinning and bulging of the cornea), characterized by progressive thinning and protrusion of the cornea, similar to keratoconus [2], as well as corneal infection [3]. The incidence of post-RK ectasia remains uncertain; however, it is estimated to be relatively rare. A multifactorial etiology may underlie the development of ectasia following RK, including, but not limited to, preexisting undiagnosed keratoconus, biomechanical weakening from the incisions, and the natural biomechanical changes that occur with age [4].

The management of post-RK ectasia presents a unique set of challenges due to the compromised corneal architecture. Potential treatment options include conservative measures such as rigid gas-permeable contact lenses; minimally invasive interventions like corneal collagen cross-linking (CXL) [5]. In advanced cases, surgical interventions such as deep anterior lamellar keratoplasty (DALK) or PKP may be considered [6].

The following case report presents a case of unilateral corneal ectasia with acute hydrops (sudden accumulation of fluid in the cornea resulting from a break in Descemet's membrane) that developed 34 years after bilateral RK. The clinical presentation, management, and review of current treatment options for this condition are highlighted.

## Case presentation

A 52-year-old male patient was referred to our ophthalmology department in April of 2022, presenting with a chief complaint of a decline in visual acuity in both eyes, particularly in the right eye. His medical history revealed a bilateral RK procedure that was performed in 1990 when he was 20 years old. Prior to the current presentation, the patient had noticed a gradual decline in vision over the previous few years, with more rapid deterioration in the right eye.

At the time of his initial visit, his best-corrected visual acuity (BCVA) was 0.04 decimal (approximately 20/500) in the right eye with manifest refraction of S±0.00D C-5.00D Ax90°, and 0.6 decimal (approximately 20/33) in the left eye with manifest refraction of S+3.00D C-2.50D Ax150°. Intraocular pressure (IOP) was measured at 7.0 mmHg in the right eye and 24.3 mmHg in the left eye.

**Figure 1:**
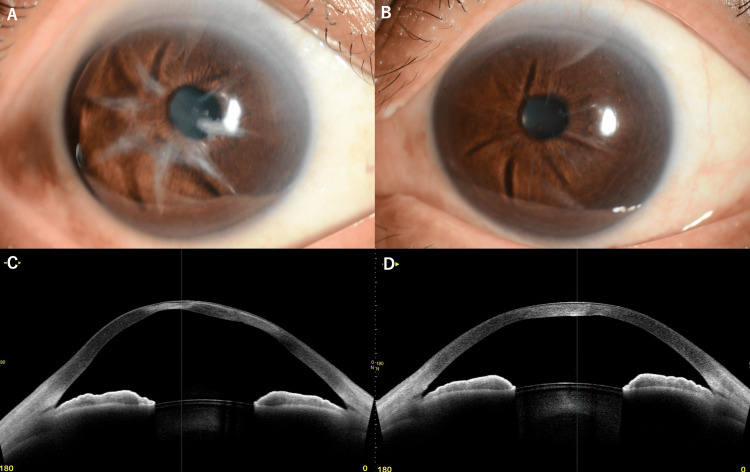
Comparison of bilateral eyes at initial presentation (A) Right eye showing significant wide corneal opacity along radial keratotomy incisions. (B) Left eye showing only mild corneal changes. (C) Anterior segment optical coherence tomography (AS-OCT) horizontal cross-section (3 to 9 o'clock meridian) of the right eye demonstrating significant corneal thinning and ectasia with a central corneal thickness (CCT) of 357 μm. (D) AS-OCT horizontal cross-section (3 to 9 o'clock meridian) of the left eye showing preserved corneal architecture without significant thinning with minimal opacity and a normal CCT of 559 μm.

A slit-lamp examination revealed significant wide corneal opacity along the RK incision sites in the right eye, while the left eye showed only mild corneal opacity. Anterior segment optical coherence tomography (AS-OCT) (CASIA2; Tomey Corporation, Nagoya, Japan) [7] revealed marked corneal thinning in the right eye, with a central corneal thickness (CCT) of 357 μm, as compared to 559 μm in the left eye, which exhibited no thinning (Figures 1A-1D).

**Figure 2:**
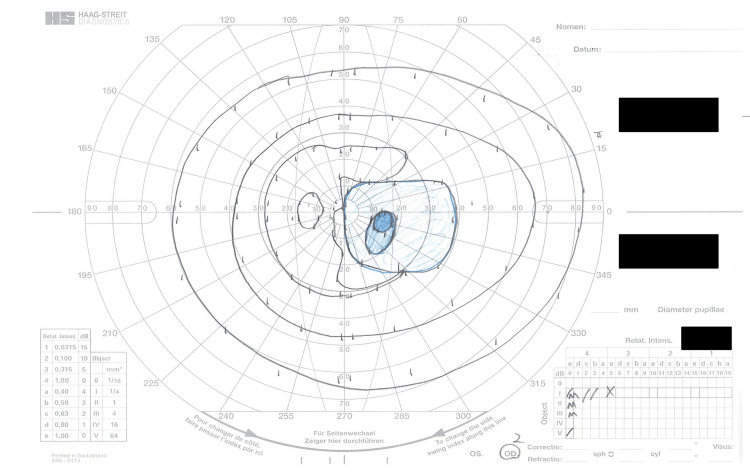
Goldman perimetry visual field test results of the right eye. The blue-shaded area represents the patient's visual field with a distinct central scotoma. Concentric circles indicate different isopters measured at various stimulus intensities and sizes, showing the extent and pattern of the central visual field defect.

Further diagnostic procedures, including retinal optical coherence tomography (OCT), revealed retinal thinning consistent with myopic fundus in both eyes but no evidence of macular pathology such as choroidal neovascular membrane, lacquer cracks, or foveoschisis. Visual field testing revealed a central scotoma in the right eye (Figure 2).

Following consultation with the glaucoma team, glaucoma was ruled out as a cause of the visual field defect. In light of these findings and after a comprehensive glaucoma assessment (including gonioscopy revealing open angles, intraocular pressure measurements, evaluation of ganglion cell complex (GCC) thickness on OCT, and correlation of optic nerve appearance with visual field defects), glaucoma was ruled out as a cause of the central scotoma. Given the poor visual prognosis, corneal transplantation was not initially recommended, and observation was chosen as the initial management approach.

**Figure 3:**
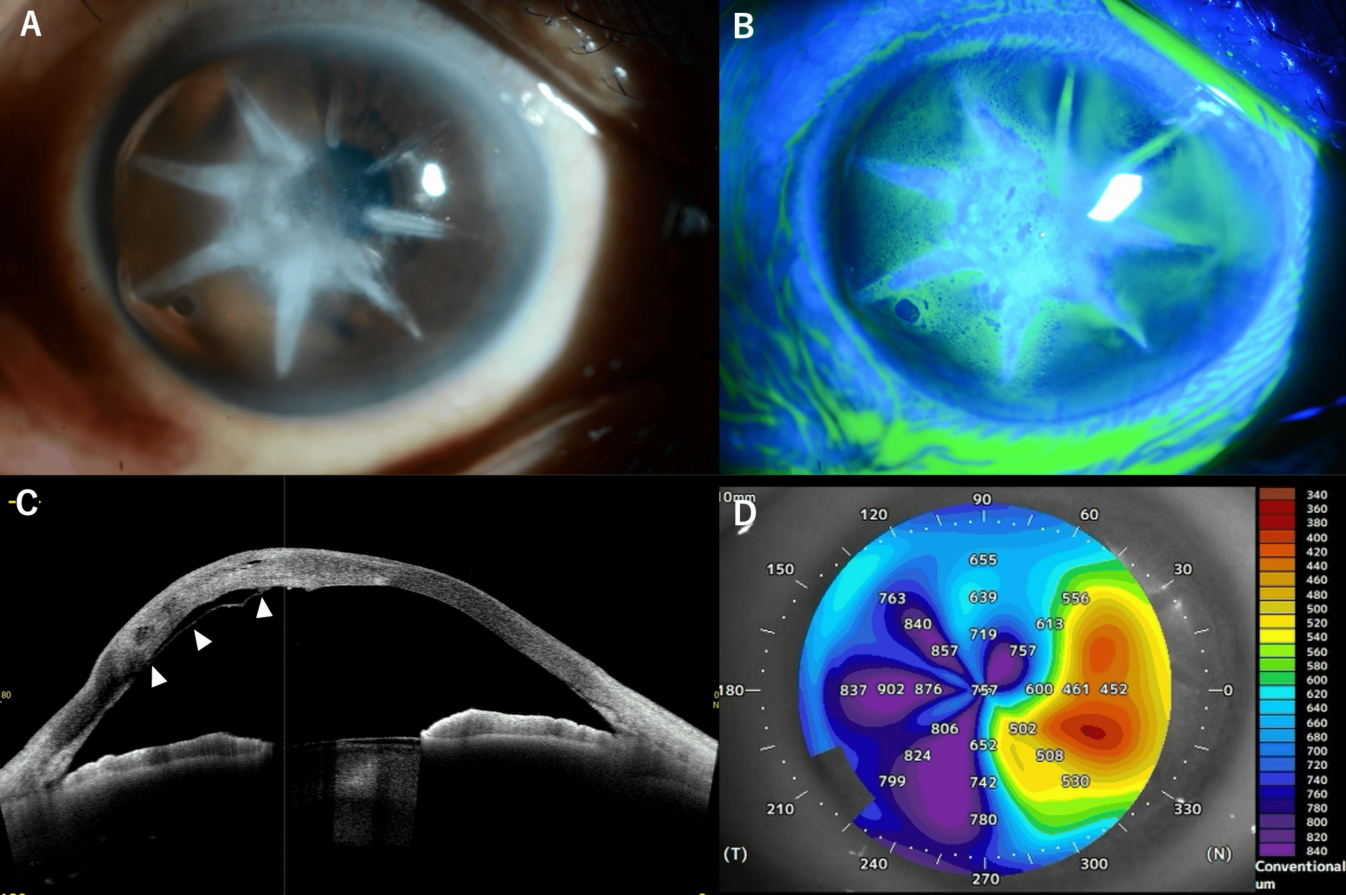
Imaging of right eye with acute hydrops occurring two years following initial presentation (A) Slit-lamp photograph showing corneal edema along the radial keratotomy incisions. (B) Fluorescein staining highlighting the areas of epithelial disruption along the incision lines. (C) Anterior segment optical coherence tomography horizontal cross-section (3 to 9 o'clock meridian) showing significant corneal edema with Descemet's membrane rupture (white arrowheads). (D) Corneal pachymetry map demonstrating increased corneal thickness (757 μm centrally) due to acute hydrops.

Two years after the initial presentation, when the patient was 54 years old, he returned with worsening symptoms in his right eye. Examination showed the corneal opacity and edema along the mainly temporal RK incisions had worsened. The BCVA remained poor at 0.05 decimal (approximately 20/400) in the right eye with manifest refraction of S±0.00D C-5.00D Ax90°. We diagnosed the patient with acute hydrops because of corneal edema and Descemet's membrane rupture in the right eye. The CCT had increased to 757 μm due to the edema (Figure 3A-3D). The patient was treated with topical gatifloxacin and fluorometholone four times daily.

**Figure 4:**
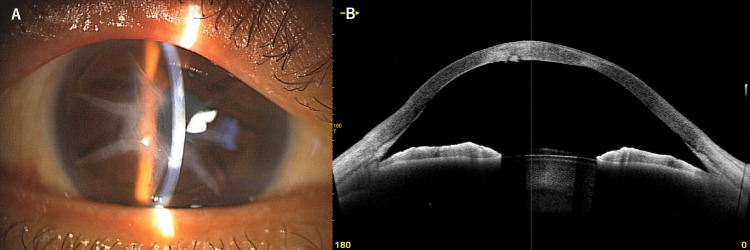
Clinical findings in post-recovery phase three months after acute hydrops in a patient with post-radial keratotomy (RK) ectasia (A) Slit-lamp photograph of the right eye showing RK incisions and partially resolved corneal opacity after acute hydrops. (B) Anterior segment optical coherence tomography horizontal cross-section (3 to 9 o'clock meridian) of the right eye demonstrating corneal thickening (central corneal thickness: 661 μm) with resolved Descemet's membrane rupture that was present during the acute hydrops phase.

Over the following months, the corneal edema improved gradually with conservative management. At 3 months follow-up after acute hydrops onset, the BCVA in the right eye had improved to 0.15 decimal (approximately 20/133) with manifest refraction of S+1.00D C-7.00D Ax90°. (Figure 4A, 4B).

## Discussion

This case underscores salient features of post-RK ectasia. First, the unilateral nature of severe ectasia in this patient is noteworthy. Despite the simultaneous bilateral RK procedure, the development of significant ectasia with acute hydrops was observed in the right eye, while the left eye exhibited relatively good vision with only mild corneal changes. This asymmetric progression suggests that factors beyond the RK procedure itself may contribute to ectasia development, such as eye rubbing, individual variations in corneal biomechanics, or possibly undiagnosed subclinical keratoconus prior to RK [8]. This asymmetric progression may not only reflect individual variations in corneal response to RK, but could also suggest that our understanding of post-RK ectasia as primarily a procedure-related complication may require reconsideration in some cases.

Secondly, the protracted period (32 years) between RK and the manifestation of symptomatic ectasia detected at the initial examination underscores the necessity for lifelong monitoring of patients who have undergone RK. While the average reported time to ectasia development after refractive surgery is approximately 15 months, cases occurring many years later have been documented [9]. This delayed presentation may be attributed to the gradual weakening of RK incisions over time, in conjunction with age-related changes in corneal biomechanics.

Thirdly, the occurrence of acute hydrops in this patient is a clinical feature that is typically associated with keratoconus but can also occur in post-RK ectasia. The rupture of Descemet's membrane precipitates acute stromal edema as aqueous humor infiltrates the corneal stroma. This advanced stage of ectasia underscores the shared pathophysiology between post-RK ectasia and keratoconus [10].

A multitude of management approaches for post-RK ectasia have been delineated in the extant literature. Contact lenses, particularly rigid gas permeable or scleral lenses, have been employed to achieve visual rehabilitation in mild to moderate cases [11]. For patients experiencing progressive ectasia, CXL has demonstrated efficacy in halting progression in cases of post-LASIK ectasia and may hold promise for post-RK ectasia as well [5, 12]. However, it is imperative to exercise caution, as CXL has been observed to exert stress on the RK incisions, which may potentially exacerbate the condition in certain cases. Literature reviews suggest that CXL may stretch the wound lips of RK, possibly preventing the cross-linking effect from reaching the pathological area and potentially resulting in new onset instability [13]. Furthermore, it is imperative to exercise caution regarding treatment selections. According to the findings of Kugler et al. [14], adding an intrastromal corneal ring segment (ICRS) should be avoided in cases of eyes that have undergone RK or other incisions that may overlie the ring segment. A comprehensive review of corneal melt cases following ICRS implantation revealed that among 1,835 eyes, 12 cases of corneal melt were documented, amounting to 0.7% of the total cases. Notably, the majority of these cases (58%) were associated with overlying incisions. In cases where ICRS is deemed unavoidable, the researchers propose the surgical closure of overlying wounds to mitigate the potential for gape and subsequent complications, though this approach should be considered experimental and currently limited to specialized centers with appropriate expertise [14].

In cases of significant corneal thinning, DALK has been shown to preserve the host endothelium while replacing the anterior corneal stroma. The "tuck-in" technique for DALK has demonstrated encouraging outcomes in managing post-RK ectasia, achieving visual enhancement, and restoring peripheral corneal thickness with minimal risk of RK incision dehiscence [6]. However, if DALK becomes technically challenging or impossible due to complications during the procedure, PKP should be considered as a backup option. For cases of severe scarring or hydrops, penetrating keratoplasty PKP may be indicated, though it carries higher risks of rejection and complications compared to lamellar techniques [15]. 

In the case presented, the patient exhibited a central scotoma of unknown origin, which introduced a complex layer of intricacy to the management strategy. Despite the resolution of corneal haziness following the resolution of the hydrops, the underlying scotoma is likely to constrain visual potential, irrespective of corneal interventions. This underscores the necessity for a comprehensive evaluation of all potential causes of vision loss prior to undertaking invasive procedures.

For this complex case, our initial management approach of observation was justified by several key factors: the presence of an unexplained central scotoma that would likely limit visual potential regardless of corneal intervention; the pronounced asymmetry between eyes with preserved visual acuity (BCVA 20/33) in the left eye; the encouraging improvement of BCVA from 20/400 to 20/133 in the right eye after conservative treatment of acute hydrops; and the significant risks associated with invasive procedures such as corneal transplantation in eyes with previous RK incisions, which may not be warranted given the uncertain visual prognosis due to the preexisting scotoma. This conservative approach allows for monitoring of disease progression while avoiding surgical risks in a case with complex visual pathology.

The asymmetric presentation in this patient gives rise to the question of whether the contralateral eye might develop similar changes in the future. Consequently, close monitoring of the left eye is imperative, as early intervention might avert the progression to acute hydrops if ectatic changes begin to manifest.

## Conclusions

Post-RK corneal ectasia is a rare, sight-threatening complication that can occur decades after the initial procedure. It presents as acute hydrops resembling keratoconus, as in this case, and is an advanced stage of the condition. This case emphasizes the importance of systematic surveillance, including annual or more frequent corneal topography and pachymetry for all post-RK patients over 40 years of age. Clinicians should be vigilant for asymmetric changes between eyes and educate patients about avoiding eye rubbing and recognizing vision changes. Management decisions should be individualized based on the severity of ectasia, the presence of other pathologies, and the patient's visual needs. Research is needed to better understand the long-term changes that occur after RK and to develop optimal strategies for preventing and managing post-RK ectasia.
